# Epidemiological study and genetic characterization of inherited muscle diseases in a northern Spanish region

**DOI:** 10.1186/s13023-019-1227-x

**Published:** 2019-12-02

**Authors:** Inmaculada Pagola-Lorz, Esther Vicente, Berta Ibáñez, Laura Torné, Itsaso Elizalde-Beiras, Virginia Garcia-Solaesa, Fermín García, Josu Delfrade, Ivonne Jericó

**Affiliations:** 1 Department of Neurology, Complejo Hospitalario de Navarra, IdiSNA (Navarre Institute for Health Research), Pamplona, Spain; 2 Community Health Observatory Section, Instituto de Salud Pública y Laboral de Navarra, IdiSNA, Pamplona, Spain; 3 Department of Health Sciences, Universidad Pública de Navarra (UPNA), IdiSNA, Pamplona, Spain; 4 Methodology Unit. Navarrabiomed, Universidad Pública de Navarra (UPNA), IdiSNA, Pamplona, Spain; 5 Primary Care, Servicio Navarro de Salud – Osasunbidea, IdiSNA, Pamplona, Spain; 6 Miguel Servet Foundation, Navarrabiomed, Pamplona, Spain; 7 Department of Genetics, Complejo Hospitalario de Navarra, IdiSNA, Pamplona, Spain; 8 CIBER Epidemiology and Public Health (CIBERESP), Madrid, Spain; 9 Department of Neurology, Complejo Hospitalario de Navarra, 31008 Pamplona, C/ Irunlarrea Spain

**Keywords:** Inherited muscle diseases, Epidemiology, Prevalence, Neuromuscular

## Abstract

**Background:**

Inherited muscle diseases are a group of rare heterogeneous muscle conditions with great impact on quality of life, for which variable prevalence has previously been reported, probably due to case selection bias. The aim of this study is to estimate the overall and selective prevalence rates of inherited muscle diseases in a northern Spanish region and to describe their demographic and genetic features. Retrospective identification of patients with inherited muscle diseases between 2000 and 2015 from multiple data sources. Demographic and molecular data were registered.

**Results:**

On January 1, 2016, the overall prevalence of inherited muscle diseases was 59.00/ 100,000 inhabitants (CI 95%; 53.35–65.26). Prevalence was significantly greater in men (67.33/100,000) in comparison to women (50.80/100,000) (*p* = 0.006). The highest value was seen in the age range between 45 and 54 (91.32/100,000) years. Myotonic dystrophy type 1 was the most common condition (35.90/100,000), followed by facioscapulohumeral muscular dystrophy (5.15/100,000) and limb-girdle muscular dystrophy type 2A (2.5/100,000).

**Conclusions:**

Prevalence of inherited muscle diseases in Navarre is high in comparison with the data reported for other geographical regions. Standard procedures and analyses of multiple data sources are needed for epidemiological studies of this heterogeneous group of diseases.

## Background

Inherited muscle diseases (IMDs), defined as rare diseases due to their low prevalence, make up a complex group of clinically and genetically heterogeneous conditions. IMDs can appear at any age and are characterized by a variety of symptoms including progressive muscle weakness, cramps, stiffness, joint deformities, chronic pain, respiratory and/or cardiac involvement, and a broad range of cognitive impairments [1, 2]. These pathologies cause variable degrees of disability in patients and have a major impact on the quality of life and health budgets worldwide [3]. The number of subjects with this type of condition is expected to increase due to better prevention of complications and genetic diagnosis advances, thus, prevalence data are essential for future budget estimates.

Despite the relevance of identifying these data only few epidemiological studies include all types of IMDs, and their methodology and results vary widely [4–14]. There seems to be regional differences concerning the prevalence of these conditions and potential ethnic differences are not fully understood.

For comparison purposes, standardized procedures for conducting epidemiological studies in this field have been proposed [15]. In Spain, there is little published information about IMDs. Most studies have been performed at regional level, focusing on a certain type of IMD and with wide regional variations [16–19]. The aim of our study is to describe the demographic and genetic features of this group of neuromuscular diseases in Navarre for a 16-year period (2000–2015) and to estimate the prevalence according to IMD subtype, age group and geographical distribution.

### Patients and methods

Observational retrospective study based on the identification of adults and children with IMDs using all health databases available within the regional health system (from January 1, 2000 to December 31, 2015). The Navarre Ethics Research Committee approved this study. The procedures followed are in accordance with the Helsinki Declaration of 1975, as revised in 2000.

#### Study population

**Fig. 1:**
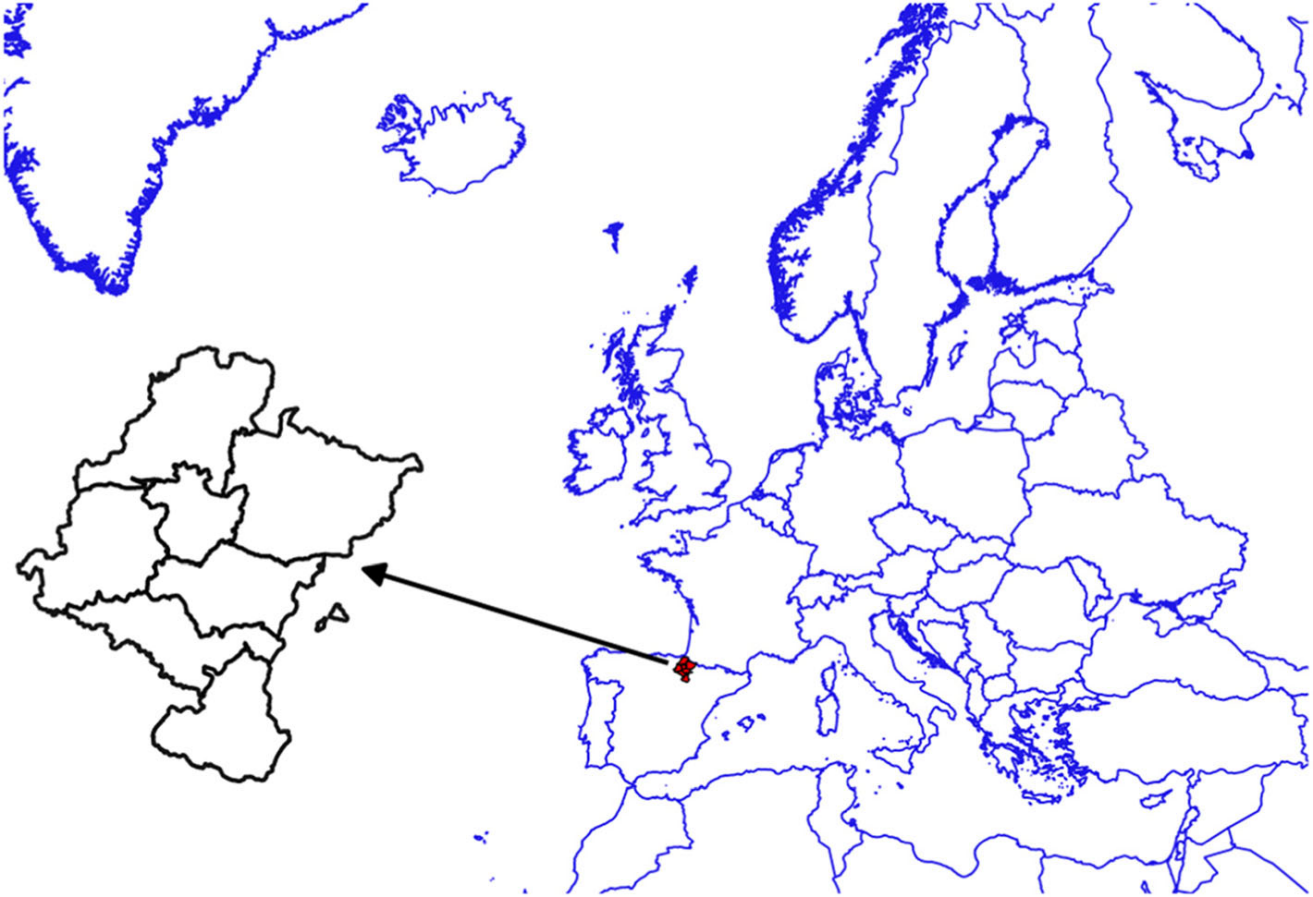
Navarre is a region in northern Spain organized in seven geographic areas

The study was conducted on a well-defined population from the Navarre Community in Northern Spain (Fig. 1) with an estimated population of 640,647 inhabitants as per the 2016 census [20]. Most citizens are covered by the Regional Public Health Service of Navarre - Osasunbidea, part of the Spanish National Health Service. Only 3.1% of the population has private o mixed health insurance [21]. Navarre is organized in seven geographic areas (Fig. 1) (Navarre 2000 Zoning) [22].

#### Diagnostic criteria

The study considered patients of any age, residents in Navarre during 2000–2015, with a definitive diagnosis or with high suspicion of suffering an IMD even without confirmed genetic diagnosis. We distinguished two groups of patients: 1) The definitive diagnosis of IMD group included subjects with genetically confirmed diagnosis as proposed in the 2017 version of the gene table of monogenic neuromuscular disorders [23] or with typical clinical phenotype consistent with a pathogenic mutation verified within the pedigree or patients with specific and well-correlated histopathological findings even in the absence of genetic confirmation. 2) The unclassified IMD group included patients with suspected but undiagnosed genetic muscle disease according to the phenotypes described by Harris et al. [24] following a thorough analysis of the patient: a) congenital onset and normal or mildly elevated creatine kinase (CK) levels; b) adult onset proximal weakness with significantly elevated CK and possible recessive inheritance; c) myopathy with prominent contractures. Patients from the second group did not meet the criteria of definitive IMD subtype as shown in Table 1. Subjects with muscle channelopathy, mitochondrial myopathies, female carriers of dystrophinopathy or isolated hyperCKemia were excluded from this study.

#### Genetic analysis

Blood was collected from patient after obtaining informed consent. DNA was extracted using standard procedures from peripheral blood samples taken from all patients. Appropriate genetic studies were performed in each case.

Sequencing techniques after amplification of all coding exons and adjacent areas of different genes associated to inherited muscle disease (*CAPN3, DYSF, SGCG, SGCA, FKRP, ANO5, PABPN1, EMD, LMNA, GMPPB, GAA, PYGM, CPT2, MYH-7, ACTA1, LDB3*) were performed to determine the DNA variants consistent in base changes; substitutions, and small insertions and deletions. First studies were carried out by Sanger sequencing following diagnostic algorithms gene-to-gene, while the implementation of the next generation sequencing (NGS) techniques in clinical diagnosis was studied by groups or panels in different NGS platforms. Bioinformatic tools were used to the alignment of the sequences to human reference genome; and detection, annotation and prioritization of variants.

*DMD* gene dosage analysis was determined by multiplex ligation-dependent probe amplification (MLPA). The SALSA® MLPA® P034 DMD-1 and P035 DMD-2 (MRC-Holland, Amsterdam) were used for the detection of exon deletions or duplications in the *DMD* gene while point mutations were identified by sequencing studies.

Myotonic dystrophy type l (DM-1) is caused by (CTG)n repeat expansion in the 3′-untranslated region of the *DMPK* gene. The sizing of this expansion was done by conventional PCR, fragment-length analysis, repeat-primed PCR, and fragment-length analysis.

The contraction of the D4Z4 repeat on chromosomes 4 is responsible of the facioscapulohumeral muscular dystrophy type 1 (FSHD1). The size of the D4Z4 repeats was determined by pulsed field gel electrophoresis (PFGE) as previously described [33]. Facioscapulohumeral muscular dystrophy type 2 (FSHD2) was studied by sequencing of *SMCHD1* gene, which is involved in the maintenance of D4Z4 methylation.

Variants of interest detected by sequencing were classified according to different databases and the published literature. Population databases: 1000 Genomes Project (http://browser.1000genomes.org), Exome Variant Server (http://evs.gs.washington.edu/EVS) and Exome Aggregation Consortium (http://exac.broadinstitute.org/). Disease databases: Human Gene Mutation Database (http://www.hgmd.org), Leiden Open Variation Database (http://www.lovd.nl) and ClinVar (http://www.ncbi.nlm.nih.gov/clinvar). In silico approach was carried out to assess the pathogenicity of new variants using different tools as Mutation Taster (http://www.mutationtaster.org).

Methodological validation and segregation studies were performed by direct sequencing (ABI 3500 Genetic Analyzer, Applied Biosystems, Warrington, UK) using Big Dye Terminator Cycle Sequencing Kit (Applied Biosystems, Warrington, UK). The subsequent analysis was done with SeqScape software (Thermo Fisher).

#### Case ascertainment sources

Case ascertainment was achieved using multiple overlapping sources:

(1) Navarre’s Minimum Basic Data Set (MBDS), a regional computer-based database system designed to collect demographic, clinical and administrative data on discharges, including both private and publicly funded hospitals. International Classification of Diseases (Ninth Revision, Clinical Modification, ICD-9-CM), including 271.0, 272.7, 359.0, 359.1, 359.2, 359.21, 359.22, 359.29, 359.89 and 359.9, were used to search patients affected by IMDs [34].

(2) Electronic Clinical Records in Primary Care (ECRPC) of Navarre‘s Public Health System, a regional healthcare information system that allows registering demographic, clinical and administrative data on primary care episodes. International Classification of Primary Care, second edition (ICPC-2) was used to encode healthcare episodes [35]. In Navarre, the ECRPC system proposes several literal descriptors linked to the ICPC-2 codes for general practitioners, including muscular dystrophy and unspecified myopathy for code N99 (Neurological disease, other), which we used for selecting the patients [36].

(3) Temporary Occupational Disability Registry of Navarre, designed to collect data on sick leaves. ICD-9-CM codes, including the aforementioned ones, were used to select patients affected by IMDs [36].

(4) Records from the Medical Genetics Service allowed selecting patients that were being monitored for IMD suspicion. Several keywords enabled us to detect these patients, depending on the reason for the request of the genetic study.

(5) The Congenital Anomalies and Hereditary Diseases Registry of Navarre, a population-based listing affiliated member to EUROCAT [37]. Keyword diagnostic searches were used to select patients suffering IMDs.

(6) Navarre’s Mortality Statistics in which the primary cause of death from the Medical Death Certificate [38, 39] is listed. We used the ICD-10 codes, including G71.0, G71.1, G71.2, G71.3, G71.8, G71.9, G72.8, G72.9 and G73.6 to identify IMD patients.

(7) Electronic Clinical Records from the Neurology Services of Navarre’s public hospitals. Patients with IMDs were detected using keyword diagnostic searches.

The information from the various data sources showed all potential diagnoses of IMDs. This information was cross-checked with the databases for duplication. Next, a neuromuscular neurologist verified the diagnosis of each double-checked case based on the inclusion and exclusion criteria for each condition.

We collected the following information: type of IMD, gender, date of birth, date of death, place of residence on January 1, 2016, and genetic diagnosis.

#### Data analysis

January 1, 2016 was the date chosen for estimating prevalence. The population at risk is defined as the residents of Navarre as per the Spanish National Statistics Institute and the Statistics Institute of Navarre [20] (Navarre population = 640,647). The 2016 prevalence rates are expressed as cases/100,000 inhabitants. An overall prevalence was estimated globally and by gender, age and geographical area within Navarre. Descriptive results are presented as frequencies and proportions. Poisson distribution was assumed to estimate CI 95% for prevalence and proportions rates. For inter-group proportion comparisons, the Pearson Chi-square test was applied. Statistical analyses were conducted using the OpenEpi program [40].

## Results

**Fig. 2:**
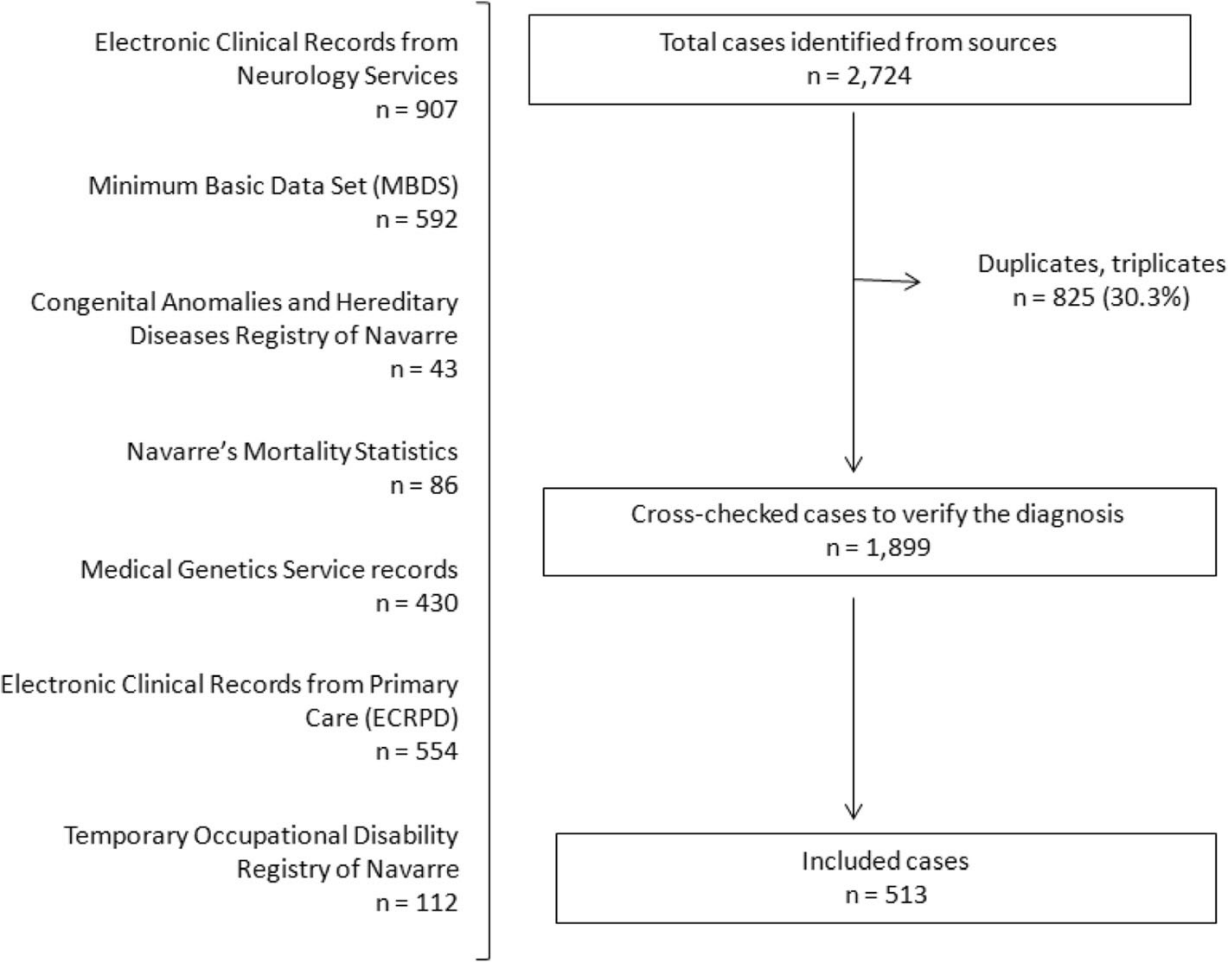
Flow-chart of potential cases of inherited muscle disease in Navarre, 2000–2015

The search strategy allowed us to retrieve 2729 potential cases after the removal of duplicates, from which 1899 required diagnostic verification (Fig. 2). Five hundred thirteen cases fulfilled the diagnostic standards listed in Table 1, representing 27.01% of the initial potential cases: 281 (54.77%) males and 232 (45.22%) females (1.21:1). Twenty-six different disease entities were detected. During the study period, 23.20% of the subjects (62 males and 57 females) died.

### Genetic and other diagnostic standards

From the 513 identified IMDs, 464 were definitive and 49 unclassified IMDs, respectively. Concerning the definitive IMD cases, 329 (70.90%) were genetically verified, 113 (24.35%) had a clinical phenotype with a pathogenic mutation confirmed with the pedigree, and in 22 (4.74%), characteristic muscle biopsy pathogenic features were found (Table 2).

**Table 1:** Diagnostic criteria used for each Inherited Muscle Disease in our study

Hereditary muscle disease type	Diagnostic criteria
Muscular Dystrophy	
Myotonic dystrophy types 1 and 2	Genetic confirmation or, characteristic clinical phenotype + a pathogenic mutation confirmed within the pedigree
FSHD, LGMD, OPMD, EDMD	Genetic confirmation or, characteristic clinical phenotype + a pathogenic mutation confirmed within the pedigree
Dystrophinopathies	Genetic confirmation or,
DMD	characteristic clinical phenotype + absence of dystrophin in Western blot
CMD	Genetic confirmation or,
Dystroglycanopathies	characteristic clinical phenotype + muscle biopsy with loss of α-dystroglycan [25]
Unclassified	characteristic clinical phenotype with onset < 2 years + muscle biopsy with dystrophic pattern
Metabolic Myopathies	
Glycogen storage disease	Genetic confirmation or,
GSD-V	characteristic clinical phenotype + increased serum CK + muscle biopsy with vacuoles with glycogen deposition and absence of myophosphorylase activity [26]
Unclassified	Characteristic clinical phenotype + increased in serum CK + muscle biopsy with glycogen deposition
Disorders of glycogen degradation	Genetic confirmation
Lipid storage disease	Genetic confirmation
Congenital myopathies	
Central core	Genetic confirmation or, clinical phenotype + muscle biopsy with cores with devoid of oxidative enzyme activity and type 1 fibre predominance [27]
Centronuclear	Genetic confirmation or, clinical phenotype + muscle biopsy with central nuclei [28]
Myosin storage myopathy	Genetic confirmation or, clinical phenotype + muscle biopsy with sarcomeric aggregation of myosin rod filaments [29]
Nemaline myopathy	Genetic confirmation or, clinical phenotype + muscle biopsy with rod-like structures in muscle fibres [30]
Fibre type disproportion	Genetic confirmation or, clinical phenotype + muscle biopsy with type 1 fibre diameter at least 35–40% smaller than type 2 fibres diameter in the absence of other structural abnormalities [31]
Myofibrillar myopathies	Genetic confirmation
Distal myopathies	Genetic confirmation or, clinical phenotype + myopathic findings on muscle biopsy + myopathic findings on electromyography + magnetic resonance imaging patterns [32]
Unclassified myopathies	Congenital onset and normal or mildly elevated CK levels or, adult onset proximal weakness + significantly elevated CK and possible recessive inheritance or, myopathy + prominent contractures

*FSHD* Facioscapulohumeral muscular dystrophy, *LGMD* Limg girdle muscular dystrophy, *OPMD* Oculopharyngeal muscular dystrophy, *EDMD* Emery-Dreifuss muscular dystrophy, *DMD* Duchenne muscular dystrophinopathy, *CMD* Congenital muscular dystrophy

Genetic descriptions of dystrophinopathies are detailed in Table 3, while the rest of IMDs can be seen Table 4**.**

**Table 2:** Inherited Muscle Disease subtype and diagnostic standard used

HM Type	Diagnostic standard	N° of cases
DM-1	Genetic confirmation	225
	Clinical phenotype + pedigree	97
FSHD	Genetic confirmation	32
	Clinical phenotype + pedigree	9
LGMD2	Genetic confirmation	27
	Clinical phenotype + pedigree	4
LGMD2C	Clinical phenotype + muscle biopsy	2
OPMD	Genetic confirmation	5
EDMD	Genetic confirmation	5
DMD	Genetic confirmation	13
	Clinical phenotype + muscle biopsy	2
BMD	Genetic confirmation	7
Dystroglycanopathies	Genetic confirmation	1
	Clinical phenotype + pedigree	1
	Clinical phenotype + muscle biopsy	1
Unclassified CMD	Clinical phenotype + muscle biopsy	2
GSD-II	Genetic confirmation	2
GSD-V	Genetic confirmation	4
	Clinical phenotype + muscle biopsy	2
Unclassified GSD	Clinical phenotype + muscle biopsy	2
Lipid storage disease	Genetic confirmation	1
Central Core	Clinical phenotype + muscle biopsy	2
Myosin storage myopathy	Genetic confirmation	2
	Clinical phenotype + muscle biopsy	1
Nemaline myopathy	Genetic confirmation	1
	Clinical phenotype + muscle biopsy	2
Fibre type disproportion	Clinical phenotype + muscle biopsy	3
Zaspopathy	Genetic confirmation	4
	Clinical phenotype + pedigree	2
Distal myopathies	Clinical phenotype + magnetic resonance imaging patterns	3

*DM-1* Myotonic dystrophy type 1, *FSHD* Facioscapulohumeral muscular dystrophy, *LGMD* Limb girdle muscular dystrophy, *OPMD* Oculopharyngeal muscular dystrophy, *EDMD* Emery-Dreifuss muscular dystrophy, *DMD* Duchenne muscular dystrophinopathy, *CMD* congenital muscular dystrophy*, BMD* Becker muscular dystrophinopathy, *CMD* Congenital muscle dystrophy, *GSD* Glycogen storage disease.

**Table 3:** Genetically confirmed dystrophinopathies

Case number	Dystrophinopathy type	Gene	Mutation type	Position and/or sequence variation
1	DMD	*DMD*	Deletion	Exons 44–55
2	DMD	*DMD*	Deletion	Exons 18–28
3	DMD	*DMD*	Duplication	Exons 18–48
4	DMD	*DMD*	Deletion	Exons 49 y 50
5	DMD	*DMD*	Deletion	Exons 45–53
6	DMD	*DMD*	Deletion	Exons 45–49
7	DMD	*DMD*	Duplication	Exon 3
8	DMD	*DMD*	SNV	c.353G > A, p.Trp118^a^
9	DMD	*DMD*	Deletion	Exon 43
10	DMD	*DMD*	Deletion	Exons 44–50
11^a^	DMD			
12^a^	DMD			
13^b^	DMD			
14	BMD	*DMD*	Deletion	Exon 52
15	BMD	*DMD*	Deletion	Intron 49
16	BMD	*DMD*	Deletion	Exons 3–7
17	BMD	*DMD*	Deletion	Exons 45–55
18	BMD	*DMD*	Duplication	Exon 2
19^b^	BMD			
20^b^	BMD			

^a^No mutation identified. Negative deletion/duplication study. Positive familial segregation

^b^Evidence of genetic confirmation in the clinical record; no access to the identified mutation

**Table 4:** Pathogenic molecular defect of each genetically confirmed IMD

Muscular disease type	Gene	Mutation type	Sequence variation	Position	Zygosity	Cases, n	Families, n
Muscular dystrophy							
MD1	DMPK	Expanded CTG (> 40)		3′-UTR		225	116
FSHD1	DUX	Deletion D4Z4				25	21
FSHD2	SMCHD1	SNV	c.5602C > T	Exon 45	Het	6	1
	SMCHD1	SNV	c.2329A > T	Exon 18	Het	1	1
LGMD2A	CAPN3	Frameshift variant	c.2362_2363delinsAG/TCATCT	Exon 22	Hom	16	15
	CAPN3 CAPN3	SNV Frameshift variant	c.664G > A c.2362_2363delinsAG/TCATCT	Exon 5 Exon 22	Het Het	1	1
LGMD2B	DYSF	SNV	c.895G > A	Exon 9	Hom	1	1
LGMD2C	SGCG	SNV	c.848G > A	Exon 8	Hom	2	2
LGMD2D	SGCA	SNV	c.293G > A	Exon 3	Hom	1	1
LGMD2I	FKRP	SNV	c.826C > A	Exon 4	Hom	1	1
LGMD2L	ANO5	Frameshift variant	c.1627dupA	Exon 15	Hom	1	1
	ANO5	Frameshift variant	c.191dupA	Exon 5	Hom	2	1
	ANO5 ANO5	Frameshift variant SNV	c.191dupA c.1664G > T	Exon 5 Exon 16	Het Het	1	1
	ANO5 ANO5	SNV Splice variant	c.172C > T c.1119 + 1G > T	Exon 4 Intron 12	Het Het	1	1
OPMD	PABPN1	Expanded GCN (> 10)		Exon 1		5	4
Emerin EDMD	EMD	Complete deletion				1	1
Lamin EDMD	LMNA	SNV	c.1130G > A	Exon 6	Het	2	1
	LMNA	SNV	c.215G > T	Exon 1	Het	1	1
	LMNA	SNV	c.65C > A	Exon 1	Het	1	1
DMC-Dystroglicanopathy	GMPPB	SNV	c.553C > T	Exon 5	Hom	1	1
Metabolic myopathy							
GSD-II	GAA GAA	Intronic variant SNV	c.-32-13 T > G c.1933G > T	Intron 1 Exon 14	Het Het	1	1
	GAA GAA	Intronic variant SNV	c.-32-13 T > G c.1724A > G	Intron 1 Exon 12	Het Het	1	1
GSD-V	PYGM	Stop gained	c.148C > T	Exon 1	Hom	1	1
	PYGM PYGM	Stop gained SNV	c.148C > T c.1468C > T	Exon 1 Exon 12	Het Het	1	1
	PYGM^a^					1	1
	PYGM^a^					1	1
Lipid storage disease	CPT2	SNV SNV	c.359A > G c.1547 T > C	Exon 4 Exon 4	Het Het	1	1
Congenital myopathy							
Myosin storage	MYH-7	SNV	c.5533C > T	Exon 37	Het	1	1
	MYH-7	SNV	c.1314G > A	Exon 14	Het	1	1
Nemaline	ACTA1	SNV	c.808G > C	Exon 5	Het	1	1
Myofibrillar myopathy							
Zaspopathy	LDB3	SNV	c.494C > T	Exon 5	Het	4	1

^*a*^ Evidence of genetic confirmation in the clinical record; no access to the identified mutation

*Hom* Homozygous, *Het* Heterozygous, *SNV* Single Nucleotide Variation

### Prevalence

On 1 January 2016, 378 subjects with IMDs (56.61% male and 43.39% female) were residents of Navarre, implying a prevalence of 59.00/100,000 inhabitants (CI 95%; 53.35–65.26) (Table 5). DM-1 represented the most common IMDs, with a prevalence of 35.90/100,000 (CI 95%; 31.55–40.85), followed by facioscapulohumeral muscular dystrophy (FSHD) and limb girdle muscular dystrophy 2A (LGMD2A) affecting 5.15/100,000 (95% CI; 3.67–7.23) and 2.5/100,000 (CI 95%; 1.54–4.05) inhabitants, respectively. There were 11 cases of dystrophinopathies, with Duchenne muscular dystrophy (DMD) present in 0.94/100,000 (CI 95%; 0.43–2.04) and Becker muscular dystrophy (BMD) in 0.78/100,000 (CI 95%; 0.33–1.83) individuals. Congenital myopathy, congenital muscular dystrophy (CMD), myofibrillar myopathy, and metabolic myopathy were observed in 1.25 (CI 95%; 0.63–2.46), 0.62 (CI 95%; 0.24–1.60), 0.78 (CI 95%; 0.33–1.83), and 1.71 (CI 95%; 0.95–3.07) per every 100,000 inhabitants, respectively.

**Table 5:** Inherited muscle disease prevalence in Navarre by January 1, 2016

Type of myopathy	Cases, n	PRx10^5^ (CI 95%)	PR male	PR female	p	Mean age (SD)
Muscular dystrophy	***312***	***48.70 (43.59–54.41)***	***53.80***	***43.67***	***0.066***	***46.43 (17.14)***
Myotonic dystrophy 1	230	35.90 (31.55–40.85)	36.18	35.63	0.906	47.06 (15.39)
FSHD	33	5.15 (3.67–7.23)	6.92	3.41	0.052	55.51 (14.44)
FSHD1	*27*	*4.21 (2.90–6.13)*	*5.98*	*2.48*	*0.320*	*58.15 (13.06)*
FSHD2	*6*	*0.94 (0.43–2.04)*	*0.94*	*0.93*	*0.985*	*43.67 (15.54)*
LGMD2	27	4.21 (0.90–6.13)	5.03	3.41	0.325	45.04 (17.67)
LGMD2A	*16*	*2.50 (1.54–4.05)*	*2.52*	*2.48*	*0.975*	*43.81 (14.63)*
LGMD2B	*1*	*0.16 (0.03–0.88)*	*0.31*	*0.00*	*0.496*	*56 (−)*
LGMD2C	*3*	*0.47 (0.16–1.38)*	*0.94*	*0.00*	*0.122*	*21.33 (24.45)*
LGMD2D	*1*	*0.16 (0.03–0.88)*	*0.00*	*0.31*	*0.503*	*46 (−)*
LGMD2I	*1*	*0.16 (0.03–0.88)*	*0.31*	*0.00*	*0.496*	*53 (−)*
LGMD2L	*5*	*0.78 (0.33–1.83)*	*0.94*	*0.62*	*0.675*	*59.20 (16.51)*
OPMD	2	0.31 (0.08–1.14)	0.63	0.00	0.246	72 (15,56)
EDMD	5	0.78 (0.33–1.83)	0.94	0.62	0.675	41.80 (19.32)
Emerin EDMD	*1*	*0.16 (0.03–0.88)*	*0.31*	*0.00*	*0.496*	*19 (−)*
Lamin EDMD	*4*	*0.62 (0.24–1.60)*	*0.63*	*0.62*	*0.988*	*47.5 (16.76)*
Dystrophinopathy	11	1.71 (0.95–3.07)	3.46	0.00	0.000	18.73 (18.26)
DMD	*6*	*0.94 (0.43–2.04)*	*1.89*	*0.00*	*0.015*	*8.33 (4.80)*
BMD	*5*	*0.78 (0.33–1.83)*	*1.57*	*0.00*	*0.030*	*31.2 (21.18)*
CMD	4	0.62 (0.24–1.60)	0.63	0.62	0.988	13.25 (6.34)
Glycosylation disorder	*3*	*0.47 (0.16–1.38)*	*0.63*	*0.31*	*0.616*	*10.67 (4.51)*
Unclassified CMD	*1*	*0.16 (0.03–0.88)*	*0.00*	*0.31*	*0.503*	*21 (−)*
Metabolic myopathies	***11***	***1.71 (0.95–3.07)***	***2.52***	***0.93***	***0.139***	***45.91 (19.13)***
Glycogen storage	10	1.56 (0.85–2.87)	2.52	0.62	0.062	49.10 (16.80)
GSD-II	*2*	*0.31 (0.08–1.14)*	*0.63*	*0.00*	*0.246*	*43 (12.73)*
GSD-V	*6*	*0.94 (0.43–2.04)*	*1.26*	*0.62*	*0.442*	*5.15 (20.83)*
Unclassified	*2*	*0.31 (0.08–1.14)*	*0.63*	*0.00*	*0.246*	*48 (9.90)*
Lipid storage disease	1	0.16 (0.03–0.88)	0.00	0.31	0.503	14 (−)
Congenital myopathy	***8***	***1.25 (0.63–2.46)***	***2.20***	***0.31***	***0.037***	***36.12 (14.20)***
Central Core	2	0.31 (0.08–1.14)	0.63	0.00	0.246	22.50 (24.75)
Myosin storage myopathy	3	0.47 (0.16–1.38)	0.94	0.00	0.122	44.67 (9.07)
Fibre type disproportion	3	0.47 (0.16–1.38)	0.63	0.31	0.616	36.67 (4.62)
Distal myopathy	***3***	***0.47 (0.16–1.38)***	***0.63***	***0.31***	***0.616***	***65.67 (17.78)***
Zaspopathy	***5***	***0.78 (0.33–1.83)***	***1.26***	***0.31***	***0.212***	***63.40 (4.88)***
Unclassified myopathy	***39***	***6.09 (4.45–8.32)***	***6.92***	***5.27***	***0.402***	***59.92 (21.86)***
Total	**378**	**59.00 (53.35–65.26)**	**67.33**	**50.80**	**0.006**	**46.93 (17.77)**

*SD* Standard deviation.

*OPMD* Oculopharyngeal muscular dystrophy, *EDMD* Emery-Dreifuss muscular dystrophy, *DMD* Duchenne muscular dystrophinopathy, *CMD* Congenital muscular dystrophy*, BMD* Becker muscular dystrophinopathy, *CMD* congenital muscle dystrophy, *GSD* glycogen storage disease.

The range of age was 1–89 years, with a mean age of 46.93 years (SD 17.77) (45.70 (SD 19.01) for males and 48.54 (SD 15.93) for females). The highest age-specific prevalence (Table 6) was obtained for the age range between 45 to 54 years, with a prevalence of 91.32/100,000 (CI 95%; 74.31–112.2) subjects. Prevalence was statistically significant higher in males in comparison to females for the following groups: under 15, 25 to 34, and 75 to 84 years of age.

**Table 6:** Prevalence of Inherited Muscle Disease according to group age and gender

Group age	PR/100,000 (CI 95%)	PR/100,000 (CI 95%) by gender		p
		Women	Men	
< 15	21.87 (14.44–33.11)	10.20 (4.36–23.87)	32.97 (20.59–52.80)	0.015
15–24	41.84 (28.56–61.30)	42.84 (25.04–73.29)	40.89 (23.9–69.95)	0.906
25–34	48.99 (35.39–67.82)	21.93 (11.11–43.27)	75,68 (52.37–109.40)	0.000
35–44	72.13 (57.64–90.26)	70.53 (50.95–97.62)	73.63 (54.08–100.24)	0.853
45–54	91.32 (74.31–112.2)	93.36 (69.79–124.92)	89.37 (66.81–119.6)	0.836
55–64	91.50 (72.56–115.40)	82.69 (58.58–116.7)	100.27 (73.37–137.20)	0.421
65–74	59.40 (42.92–82.23)	57.51 (36.38–90.9)	61.43 (38.86–97.09)	0.844
75–84	39.08 (24.06–63.47)	21.42 (9.15–50.14)	62.48 (34.9–111.90)	0.044
≥85	23.38 (9.99–54.73)	13.92 (3.81–50.76)	42.73 (14.53–125.62)	0.245
Total	**59.00 (53.35–65.26)**	*50.80 (43.46–59.36)*	*67.33 (58.9–76.97)*	*0.006*

**Fig. 3:**
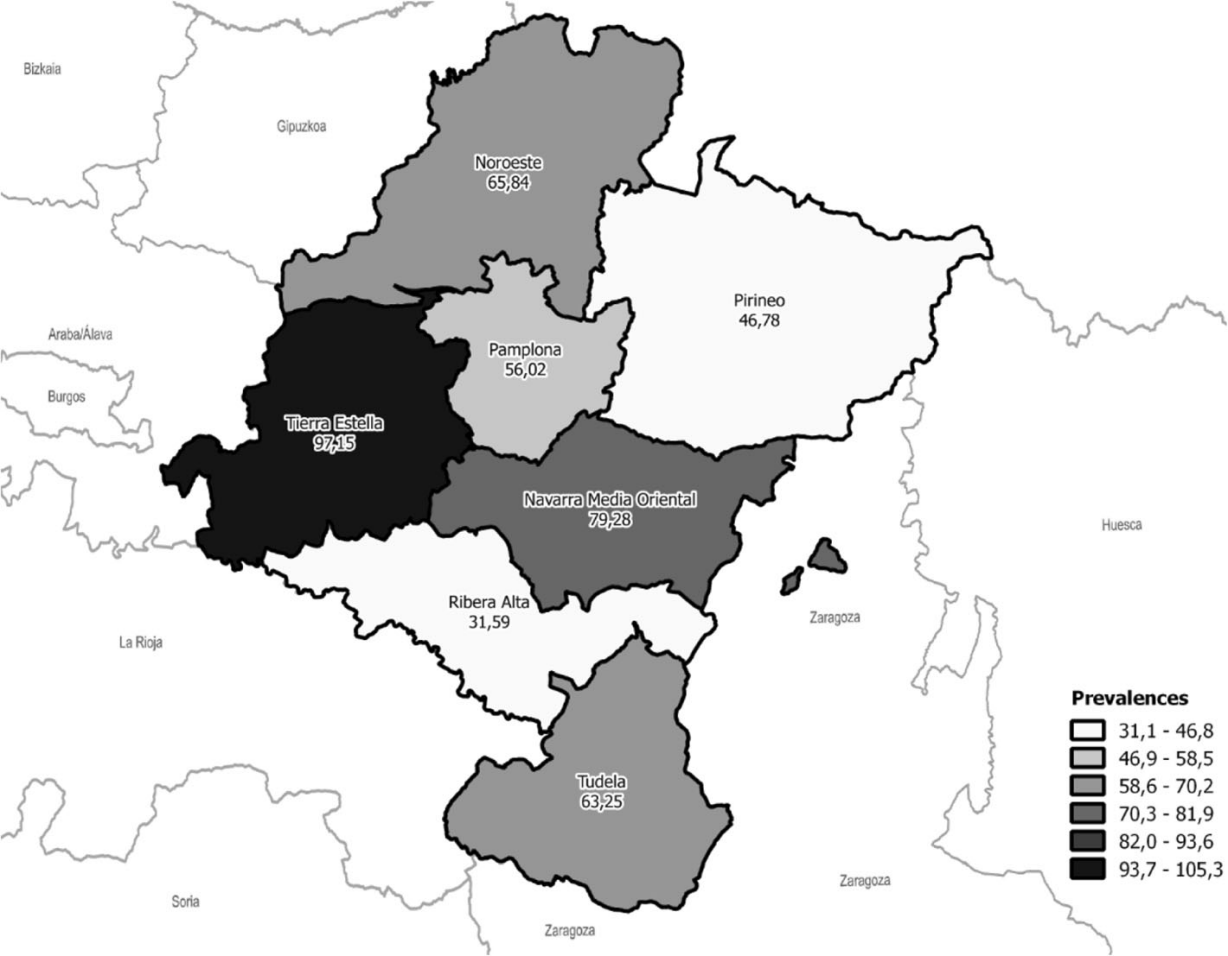
Geographical distribution of prevalence (per 100,000) of inherited muscle disease in Navarre

The prevalence of IMDs differed notably by geographic areas, with the highest estimate found for the region of Tierra Estella (97.15/100,000 subjects), significantly higher in comparison to all other areas, except for the Eastern Middle area of Navarre. Figure 3 shows the geographical distribution of IMD prevalence.

## Discussion

In this study, we present data on Inherited Muscle Diseases compiled over a 16-year period from different healthcare information systems. Our results show a prevalence of IMDs in Navarre of 59.00/100,000 subjects, being higher in males than in females, highest for the 45 to 54 years age range, and with remarkable geographical variability. DM-1, FSHD, and LGMD2A are the most common subtypes IMDs.

We believe the non-inclusion in the analyses of the unclassified group of IMDs may underestimate the real prevalence, bearing in mind that each case was thoroughly assessed by a specialized neurologist. To avoid selection biases when comparing with other studies, we also present prevalence with a confirmed genetic diagnosis: 50.10/100,000 (CI 95%; 44.92–55.89), which remains to be high in comparison to the results published elsewhere [5]. Four hundred and forty two cases (86.16%) of IMDs had a confirmed genetic diagnosis.

### Epidemiological studies of neuromuscular diseases

Prevalence studies require exhaustiveness. The lack of specific registries and the absence of diagnostic code verifications [9, 41–43] could lead an IMD selection bias [15]. Moreover, for comparability purposes stringent inclusion criteria is mandatory.

Aiming to avoid these intrinsic limitations in prevalence studies, for this study we used seven case ascertainment sources at different healthcare levels and made an exhaustive verification of the diagnoses with well-defined inclusion and exclusion criteria.

### Data sources

This study was affected by the lack of disease codification specificity in the Spanish healthcare information systems for IMDs. Only 27.01% of the cases initially identified with the selected codes and keywords met the inclusion criteria for IMD. Thus, it is essential to review and verify the diagnosis procedures to obtain quality data for this type of epidemiological.

The introduction of population-based registries specific for neuromuscular and/or other rare diseases, including specific codes, would be of great help in future studies.

### Overall and disease-specific prevalence data

Our study shows a prevalence of IMDs of 59.00/100,000 (CI 95%; 53.35–65.26) inhabitants for Navarre. Comparisons with prevalence data from other regions is complicated due to the lack of methodological homogeneity and because most studies focus on a specific IMD. Emery [44] reports a global prevalence of hereditary neuromuscular disorders of 1/3500 inhabitants (prevalence 28.57/100,000), including spinal muscular atrophy and hereditary sensitive-motor neuropathy. In another study carried out in the UK, a prevalence of IMDs of 37/100,000 people is described for a Northern region of England [6]. More recently, Theadom et al. [5] describe a prevalence of 22.3/100,000 inhabitants of all genetic muscle disorders in New Zealand, with higher incidence in subjects of European ancestry.

To the best of our knowledge, this is the first time a study includes all types of IMDs in a Spanish region.

The subtype of IMD with the highest number of cases in our series was DM-1 (58.87% of the cases) with a prevalence of 35.90/100,000 (CI 95%; 31.55–40.85). In previous works, the prevalence of this particular condition showed wide geographical variations. The lowest reported for Japan (0.2/100,000) [8] up to 172/100,000 for Quebec [45] due to the founder effect. Regarding data from Spanish regions, Burcet et al. [18] found 10.9/100,000 cases of DM-1 in Majorca, while Munain et al. [16] reported 26.5/100,000 in Guipuzcoa. These regional variations could indicate a possible underestimation of prevalence linked to the used methodology.

However, we believe that despite the used methodology, the high prevalence of DM-1 in Navarre could be explained by a possible founding effect, similar to that reported for Guipuzcoa [16], considering that both regions share cultural and background similarities. Moreover, the management of the patients by multidisciplinary teams in recent years may have a positive effect on survival by lowering the complications.

Prevalence of LGMD also differs between studies. Theadom’s review [15] reports a global prevalence of LGMD of 0.9/100,000 inhabitants. Here, we show a higher prevalence of LGMD (4.21/100,000) (CI of 95% 2.90–6.13), closer to the 4.8/100,000 estimated by Fardeu et al. [46] in a tiny community in Reunion, where high rates of endogamy have been described. In Spain, a study by Urtasun et al. in the Basque Country found a prevalence of 6.9/100,000 [17]. The most common form of LGMD is LGMD2A in the Basque Country and in our study, with over 50% of LGMD cases (59.26 and 61.29%, respectively). Both studies detected a high frequency of the c.2362_2363delinsAG/TCATCT mutation in exon 22 of the *CAPN3* gene, which has been observed primarily in chromosomes of Basque natives and more exceptionally in individuals from other parts of the world [17]. In our study, this pathogenic variant is present in 100% of the LGMD2A.

The prevalence of dystrophinopathies in our study is 0.94/100,000 for DMD and 0.78/100,000 for BMD. These values are lower than those reported elsewhere. The meta-analysis conducted by Mah et al. [47] showed an estimated prevalence of 4.78/100,000 (CI 95%; 1.94–11.81) for DMD and 1.53/100,000 (CI 95%; 0.26–8.94) for BMD. The study performed in New Zealand [5] shows a prevalence of DMD of 2.45/100,000 (CI 95%; 2.01–2.98) and 1.67/100,000 (CI 95%; 1.32–2.12) for BMD with ethnic differences. However, some studies show a prevalence of DMD below 2/100,000 [43, 48]. We believe that the poor exploitation of electronic clinical records from Paediatric Services did not cause a biased estimation of dystrophinopathies in our study.

We observed higher prevalence of IMDs in men than in women. This difference could be due to the X-linked inheritance of DMD and BMD. However, we also observed significant differences in the congenital myopathies subgroup, with higher prevalence in men. Furthermore, there was higher prevalence in men in the following age groups: under 15, 25 to 34, and 75 to 84 years of age. In the under 15 group, the X-linked nature of DMD could explain this elevated prevalence [49]. In the other two age groups, the IMD subtypes differ greatly and we have clear explanation for the gender differences. The highest prevalence of IMDs is seen for the working age group (between 35 to 64 years) probably contributing to huge socio-economic burden. Further studies should be designed to analyse the impact these conditions have on the economy.

We also detected prevalence geographical distribution differences within Navarre, which may be useful when planning resources. The highest prevalence of IMDs was determined for Tierra Estella Area (PR 97.15 with IC 95% 70.19–134.50) (Fig. 3).

### Unclassified inherited muscle disease

In the course of this study, we identified 49 patients (9.55%) with a potential genetic cause for their muscle disease. During the period of our study (2000 to 2015), most genetic diagnosis followed the gene-by-gene testing strategy based on their phenotype. Current availability of next-generation sequencing is changing the diagnostic approach, increasing confirmed genetic diagnosis, as well as the identification of new IMD-associated mutations.

Thirty-nine patients remained alive by the end of the study (December 2015) and from the end of the study to the present time genetic IMD confirmation was obtained for 18 (46, 15%).

### Study limitations

Although the study has been exhaustive, poor exploitation of the electronic clinical records in Paediatric Services could bias childhood IMD data, e.g., DMD. However, we believe that the exploitation of other data sources counteracts this deficiency, consequently with mild underestimation of IMD prevalence in this age group.

## Conclusions

The prevalence of IMDs in Navarre is 59.00/100,000 inhabitants CI (53.35–65.26), which is a high number if compared with data reported for other geographical regions. If only patients with confirmed genetic diagnosis are considered, the prevalence is 50.10/100,000 (CI 95%; 44.92–55.89). The high prevalence of DM-1 (35.90/100,000 with CI 31.55–40.85) and of LGMD2A (2.5/100,000 with CI 1.54–4.05) could suggest the existence of a founding effect in Navarre. Genetic confirmation was available in 442 (86.16%) of IMD patients in our region. Our population study has a high sensitivity because all possible sources of information have been used. The lack of specificity of disease coding in our health information system for IMDs has made the study difficult and has forced us to review the clinical data of each case to verify the diagnosis. It is essential to implement specific population based registries for neuromuscular and other rare diseases, taking into account the heterogeneity of these disorders.

## Data Availability

The data that support the findings of this study are available in the records of the health systems described in the study, but restrictions apply to the availability of these data, which were used under license for the current study, and so are not publicly available. Data are however available from the authors upon reasonable request and with permission of Complejo Hospitalario de Navarra and Navarra’s Public Health System.

## Abbreviations

BMD: Becker Muscular Dystrophy
CMD: Congenital Muscular Dystrophy
DM-1: Myotonic Dystrophy type 1
DMD: Duchenne Muscular Dystrophy
FSHD: Facioscapulohumeral Dystrophy
ICD: International Classification of Diseases
IMDs: Inherited Muscle Diseases
LGMD: Limb Girdle Muscular Dystrophy
